# Effect of Date Fruit Consumption on the Glycemic Control of Patients with Type 2 Diabetes: A Randomized Clinical Trial

**DOI:** 10.3390/nu14173491

**Published:** 2022-08-25

**Authors:** Alexandra E. Butler, Jenan Obaid, Pearl Wasif, Jean V. Varghese, Rawan Abdulrahman, Dalal Alromaihi, Stephen L. Atkin, Naji Alamuddin

**Affiliations:** 1 Research Department, Royal College of Surgeons in Ireland-Bahrain, Busaiteen 15503, Bahrain; 2 Department of Internal Medicine, King Hamad University Hospital, Busaiteen 24343, Bahrain

**Keywords:** date fruit, raisins, HbA1c, cardiovascular parameters, type 2 diabetes

## Abstract

Objective. Date fruit has been reported to have benefits in type 2 diabetes (T2D), though there is a concern, given the high sugar content, about its effects on glycemic control. Design and Setting. Prospective, interventional, randomized, parallel study. Participants. In total, 79 patients with T2D (39 male and 40 female). Intervention. Participants were randomly allocated to either 60 g date fruit or 60 g raisins daily of the equivalent glycemic index (amount split, given as midmorning and midafternoon snack) for 12 weeks. Main Outcome Measures. The primary outcome was to investigate the effect of date fruit on HbA1c and fasting blood glucose, and their variability, in patients with T2D in comparison to the same glycemic load of raisins. The secondary outcomes were to determine whether date fruit affected cardiovascular risk by measuring fasting lipids, C-reactive protein (CRP), blood pressure, and insulin resistance (IR) as measured by Homeostatic Model Assessment (HOMA-IR). Results. In total, 61 (27 female and 34 male) of 79 patients completed the study. There was no difference between or within groups for HbA1c or HbA1c variability, fasting glucose or glucose variability, insulin resistance (HOMA-IR), insulin sensitivity (HOMA-S), beta cell function (HOMA-B), the disposition index, lipids, systolic (SBP) or diastolic blood pressure (DBP), or C-reactive protein (CRP) (*p* > 0.05). Conclusion. No improvement in glycemic indices was seen following supplementation of 60 g daily date fruit or raisins, though neither had a deleterious effect on glycemic control over a 12-week period, indicating their safety when consumed in T2D. Additionally, no beneficial therapeutic effects of date fruit on other cardiovascular indices in T2D were seen.

## 1. Introduction

Date fruit has been reported to have beneficial effects due to its nutritional value and antioxidant properties [1]. However, date fruit has a high sugar content, and there is concern that heavy consumption could contribute to the increasing global prevalence of diabetes [2]. This is a particular problem in the Middle East and North Africa (MENA) region, where date consumption is high, and diabetes is an increasing health problem. According to the International Diabetes Federation, the prevalence of diabetes in the MENA region is over 23.3% [2]; globally, there is an epidemic increase in type 2 diabetes (T2D), with the World Health Organization (WHO) reporting that approximately 415 million people have diabetes worldwide and that, by 2030, diabetes will be the seventh leading cause of death.

The date palm is one of the oldest planted trees on the earth, at around 2000 years old. Dates (*Phoenix dactylifera*) are nutritionally rich and a good source of fiber and carbohydrates; their potential medicinal and nutritional effects have been suggested by a number of studies [1,3]. Date sugars have also been shown to be phenol-rich, potent antioxidants and strong inhibitors of α-glycosidase that may have benefits in diabetes [1]. In addition, dates are rich in micronutrients that may also benefit diabetes and insulin resistance [1]. Dates have a glycemic index of 50 [4], and studies have shown that the consumption of different varieties of dates does not significantly affect acute glycemia in patients with T2D [4,5,6].

Previous studies have shown that whilst the mean HbA1c may not differ between diabetes patient cohorts, the HbA1c variability is associated with both microvascular and macrovascular diabetes-related complications [7,8,9]; however, such changes have not been reported for the use of either date fruit or raisins in type 2 diabetes.

To date, there has not been an intermediate-term study on the effect of date fruit on diabetes and whether dates have specific medicinal properties. Short-term studies comparing date fruit and raisins showed no difference in the response at 30 min, confirming the equivalence in glycemic response in T2D [10] and their suitability as comparators. A comparison between date fruit and raisins allows the glycemic index to be aligned, although dates have a higher phytoestrogen content at 329 ug of phytoestrogens per 100 g, compared to raisins at 9.6 ug per 100 g [11]. It is recognized that phytoestrogens have an effect on cardiovascular indices that may underlie any effect seen with date fruit, though at much higher phytoestrogen levels than that contained within a serving of date fruit [12,13]. Date fruit has been suggested to have specific medicinal properties, and therefore, to address this, an inactive placebo with no glycemic index may give a misleading result in diabetes; thus, there is the need to account for the glycemic index in this scenario. Therefore, the objective of this study was to investigate the effect of date fruit on HbA1c and its variability, fasting blood glucose and its variability, insulin resistance (IR), insulin sensitivity (IS), beta cell function, and the cardiovascular risk indices of C-Reactive Protein (CRP) and fasting lipids in patients with T2D in comparison to raisins that have the same glycemic load.

## 2. Materials and Methods

### 2.1. Patients and Methods

King Hamad University Hospital Institutional Review Board (IRB Reference #20-344) gave approval for this study. Informed written consent was obtained from all study participants before enrolling in the trial. Inclusion criteria were patients with T2D diagnosed according to ADA criteria [14] on stable diabetes medication for 3 months prior to entry into the study, HbA1c 6.6–10%, age ≥ 21 years, and capable of providing informed consent and completing the study. Exclusion criteria included patients with HbA1c > 10% at recruitment; BMI < 20 kg/m^2^; currently taking insulin, antibiotics, or hormone replacement therapy; enrollment in another clinical trial; pregnant or nursing (or planning to become pregnant in the next 3 months); or a history of malignancy (except for non-melanoma skin cancer). Patients who were eating dates or raisins were only included if they had a washout period of 2 weeks [15].

**Figure 1:**
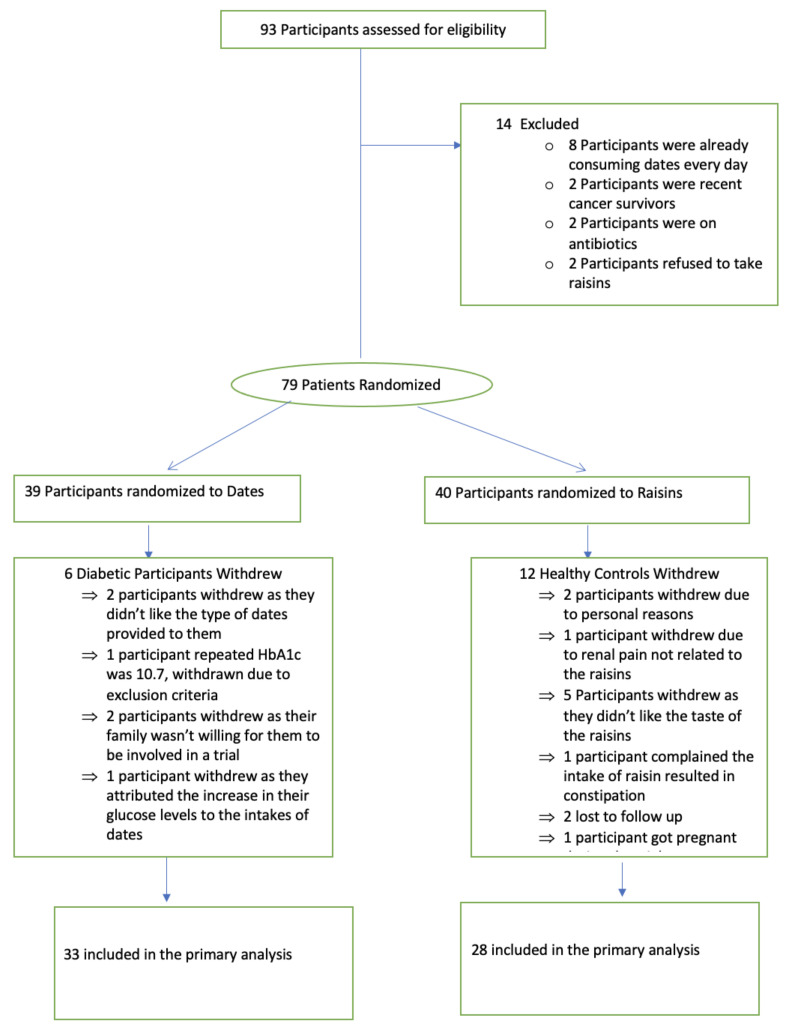
Flow diagram of the study participants.

A flow chart of study participants is shown in Figure 1. A total of 93 patients were initially identified, of whom 79 patients (39 male and 40 female) were recruited, and all were encouraged to continue their usual diet. All patients undertook the European Prospective Investigation into Cancer and Nutrition (EPIC) food frequency questionnaire (FFQ) and the International Physical Activity Questionnaire (IPAQ) short last 7-day self-administered questionnaire to assess their food intake and choices, together with their activity levels at the beginning and end of the study. The study began in October 2021 and was completed in April 2022. The study was conducted in line with the International Conference for Harmonisation of Good Clinical Practice (ICH GCP) guidelines.

### 2.2. Study Design

A randomized, parallel, interventional trial was undertaken. A total of 79 participants (age range 30–76 years; 39 males and 40 females) were block randomized; 39 patients (21 males and 18 females) were commenced on 60 g of date fruit (3 khalas dates) twice daily at midmorning and midafternoon (6 dates in total per day), and 40 patients (18 males and 22 females) were commenced on 60 g of raisins having an equivalent glycemic index (30 g midmorning and midafternoon) for 12 weeks. The study period was necessarily completed prior to the initiation of Ramadan (at which time eating patterns are markedly altered).

Participants received pre-packaged sachets of either date fruit or raisins for each individual intervention, two sachets per day for 12 weeks, plus six reserve sachets in case of study material loss.

After baseline tests, the participants were randomly allocated to either arm by a computer-generated block randomization list. Each randomization number corresponded with 1 of the 2 possible arms. Compliance was measured by counting the returned sachets, whereupon the patient had recorded their date or raisin consumption.

### 2.3. Study Measurements

Fasting venesection was undertaken at the start and finish of the 12-week study. Patients were telephoned weekly to check on compliance and to ensure safety 4-point glucose profiles were performed twice weekly and were recorded. Self-monitoring glucose devices that recorded the values were supplied for this purpose. Following an overnight fast, blood pressure (BP) and weight were measured, after which blood samples were collected. BP was measured after the participants had been seated quietly for at least 5 min with the arm supported at heart level. Fasting venous samples were collected and centrifuged at 2000× *g* at 4 °C for 15 min, and the aliquots were stored at −80 °C within 1 h of collection. Biochemical profile, HbA1c, fasting insulin, total cholesterol, triglycerides, low-density lipoprotein (LDL), high-density lipoprotein (HDL), cholesterol, and C-reactive proteins (CRP) were measured using a Synchron LX 20 analyzer (Beckman Coulter) according to the manufacturer’s recommended protocol.

The Access Ultrasensitive Insulin chemiluminescent immunoassay was used for the quantitative determination of insulin levels in human serum. The analytical sensitivity of the insulin assay was 2 μU/mL, the coefficient of variation was 6%, and there was no stated cross-reactivity with proinsulin. Plasma glucose was measured using a Synchron LX 20 analyzer (Beckman Coulter), using the manufacturer’s recommended protocol. The coefficient of variation for the assay was 1.2% at a mean glucose value of 5.3 mmol/L during the study period.

Insulin resistance was calculated using the Homeostatic Model Assessment (HOMA) method [HOMA-IR = (insulin (μU/L) × glucose (mmol/L))/22.5] [16]. Beta cell function was calculated using HOMA-B% [HOMA-B = (insulin (μU/L) × 20)/(fasting glucose (mmol/L) − 3.5)]. HOMA-S%, a measure of insulin sensitivity, was calculated as [(1/HOMA-IR) × 100]. The disposition index, a composite measure of beta cell function, was calculated as [(HOMA-S/100) × (HOMA-B/100)] [17].

All analyses were undertaken according to current guidelines, regulations, and quality control.

### 2.4. Statistical Analysis

There are currently no robust studies in man that would allow for a definitive power calculation. Probability theory from the normal distribution suggests 12–35 subjects per group for pilot randomized controlled trials (RCTs) [18,19] with power = 90% and significance = 5% (two-tailed). We went slightly above Teare’s upper estimate to allow for better precision. Recruitment of this number was doable within our timeframe. Hence, we intended to recruit 40 patients per group, which includes a 30% dropout rate (a rate commonly encountered in nutraceutical studies), therefore requiring 28 subjects per group to complete the study.

Intention to treat analysis was undertaken. Within-group differences (difference between 12-week values and baseline values) are shown for each treatment group separately by a mean and a standard deviation (SD). Data trends were visually and statistically evaluated for normality. Between-group comparisons were performed using the independent sample *t*-test. Wilcoxon’s signed-rank test was used for data that violated the assumptions of normality by the Kolmogorov–Smirnov test. Statistical analysis was performed using Graphpad Prism version 9.4.0. The data are reported as mean ± SD. We assumed an arbitrary level of 5% statistical significance (two-tailed).

Variability was calculated using a variant of the Levine test as the absolute difference from the median for each group using a Wilcoxon Mann–Whitney test that is based on the probability that an observation from one group will be farther away from the mean than an observation from the other group.

## 3. Results

The demographic and biochemical data for the patients are shown in Table 1 where it can be seen that the cohorts were well-matched for body mass index (BMI) (mean 31.3 ± 6.2 vs. 31.3 ± 5.7 kg/m^2^, dates vs. raisins), systolic blood pressure (mean 133 ± 20 vs. 133 ± 17 mmHg, dates vs. raisins), diastolic blood pressure (mean 74 ± 9 vs. 79 ± 15 mmHg, dates vs. raisins), and age (mean age 61 ± 10 vs. 56 ± 9 years, dates vs. raisins). There were no differences in baseline parameters between groups. Compliance was 98% in both groups. Of the 39 patients randomized to date fruit, 6 withdrew, and 33 (85%) completed the study; of the 40 patients randomized to raisins, 12 withdrew, and 28 (70%) completed the study (Figure 1). Weight and BMI were unchanged after either dates or raisin supplementation (Table 2).

There was no difference in total cholesterol, LDL, HDL, triglycerides, or CRP either between or within the dates and raisins groups (*p* > 0.05) (Table 2).

There were no differences between groups for both the Epic food frequency questionnaire and the IPAQ short last 7-days self-administered questionnaire that accounted for dietary changes and exercise (data are not shown, but raw data are available upon request).

**Figure 2:**
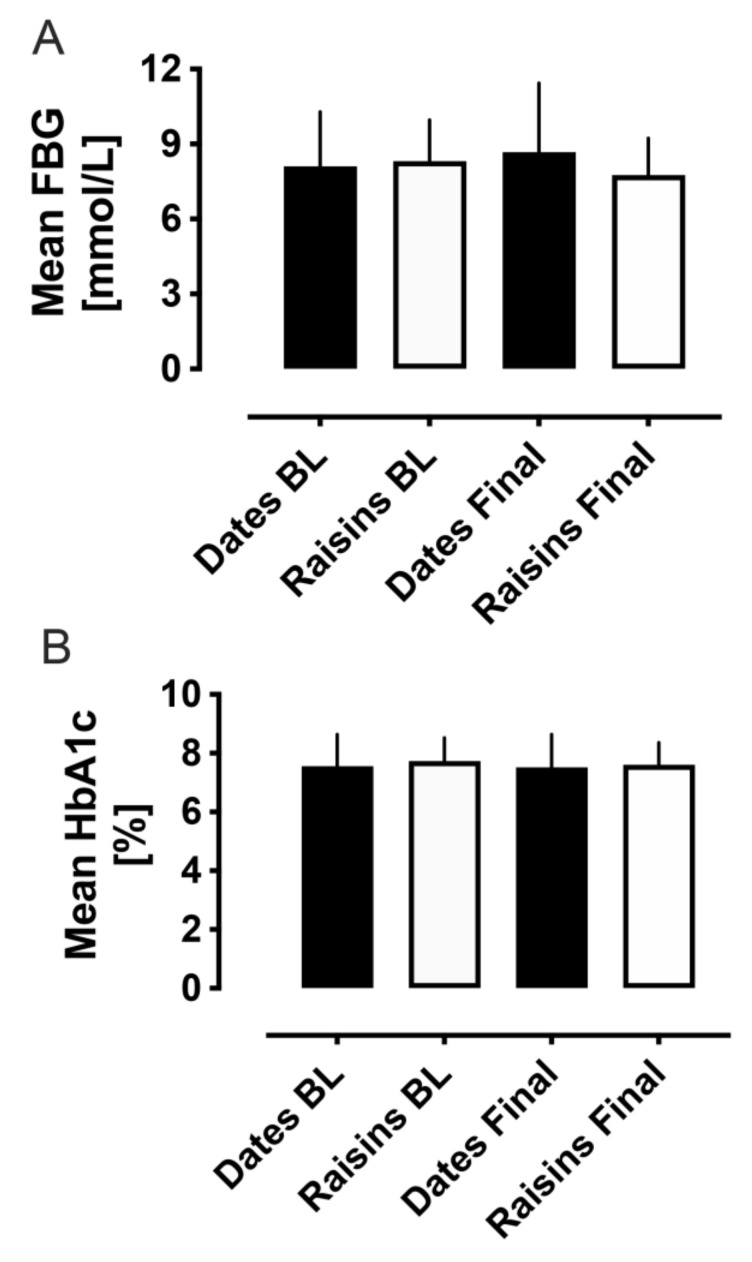
Fasting blood glucose (FBG, laboratory measurement) (**A**) and HbA1c (**B**) comparisons between baseline and at the end of the 12-week trial period. FBG and HbA1c levels showed no between or within group changes for either the date fruit or the raisins groups. BL, baseline.

Between groups, mean fasting blood glucose (FBG) did not differ at baseline or at the final visit (baseline: 8.1 ± 0.4 vs. 8.3 ± 0.3 mmol/L, dates vs. raisins, *p* = 0.69; final: 8.7 ± 0.5 vs. 7.8 ± 0.3 mmol/L, dates vs. raisins, *p* = 0.12). Neither were there any differences in FBG within groups from baseline to the final visit (dates: 8.1 ± 0.4 vs. 8.7 ± 0.5 mmol/L, baseline to final, *p* = 0.37; raisins: 8.3 ± 0.3 vs. 7.8 ± 0.3 mmol/L, baseline to final, *p* = 0.20) (Figure 2A).

The home blood glucose profiles over the 12 weeks of the study period between the baseline and final visits did not show any differences either within or between groups (Supplementary Figure S1).

Between groups, mean HbA1c did not differ at baseline or at the final visit (baseline: 7.6 ± 0.2 vs. 7.7 ± 0.1%, dates vs. raisins, *p* = 0.42; final: 7.5 ± 0.2 vs. 7.6 ± 0.1%, dates vs. raisins, *p* = 0.72), neither were there any differences within groups from baseline to the final visit (dates: 7.6 ± 0.2 vs. 7.5 ± 0.2%, baseline to final, *p* = 0.86; raisins: 7.7 ± 0.1 vs. 7.6 ± 0.1%, baseline to final, *p* = 0.51) (Figure 2B).

Between groups, HOMA-IR did not differ at baseline or at the final visit (baseline: 4.9 ± 5.1 vs. 4.2 ± 3.2, dates vs. raisins, *p* = 0.46; final: 3.8 ± 3.5 vs. 3.2 ± 1.5, dates vs. raisins, *p* = 0.42), neither were there any differences within groups from baseline to the final visit (dates: 4.9 ± 5.1 vs. 3.8 ± 3.5, baseline to final, *p* = 0.30; raisins: 4.2 ± 3.2 vs. 3.2 ± 1.5, baseline to final, *p* = 0.15). Insulin sensitivity as determined by HOMA-S, beta cell function as determined by HOMA-B, and the disposition index (a composite measure of beta cell function) did not differ either between or within groups (Table 2).

To determine variability in FBG and HbA1c, the absolute difference from the median was calculated from baseline and final visit values.

**Figure 3:**
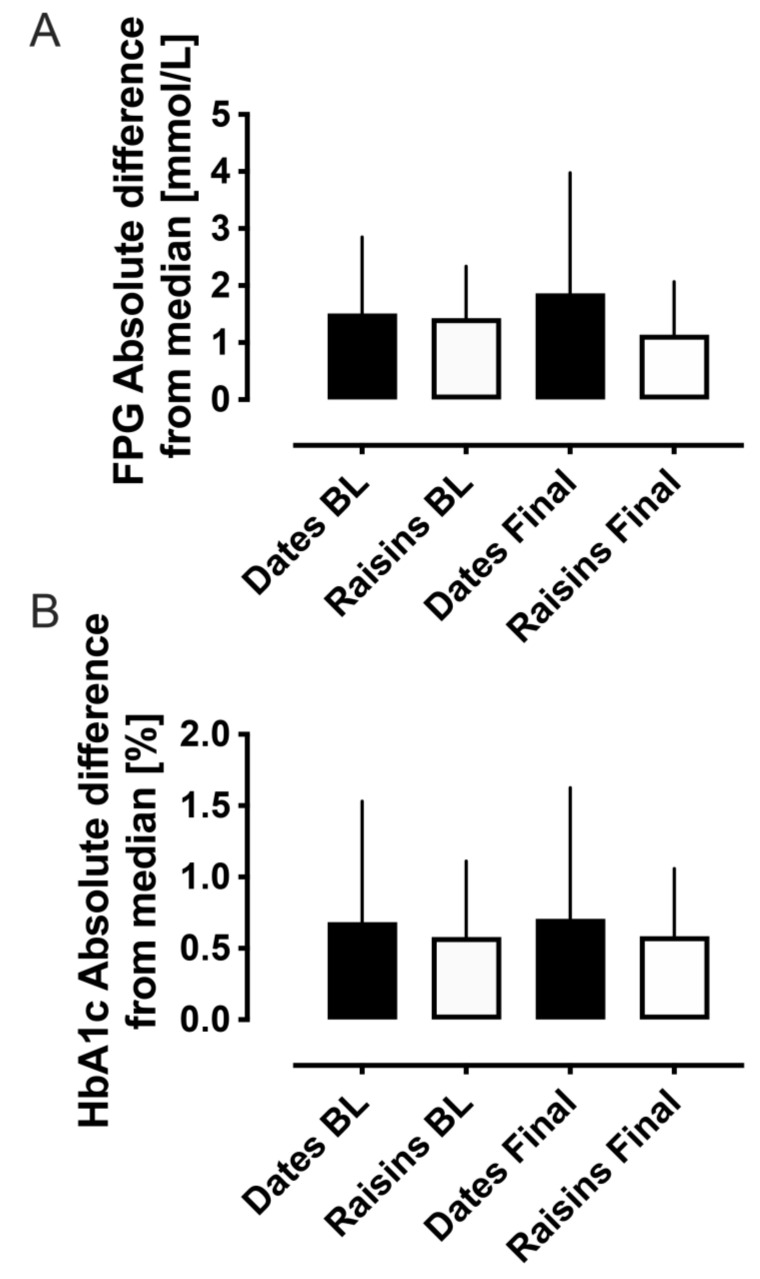
The absolute difference from the median for FPG (**A**) and HbA1c (**B**), indicating no difference in variability either between or within groups for either the date fruit or raisins. BL, baseline.

For FBG, there were no differences in the absolute difference from the median either between groups (baseline: 1.5 ± 0.2 vs. 1.4 ± 0.2 mmol/L, dates vs. raisins, *p* = 0.79; final: 1.9 ± 0.4 vs. 1.1 ± 0.2 mmol/L, dates vs. raisins, *p* = 0.23) or within groups (dates: 1.5 ± 0.2 vs. 1.9 ± 0.4 mmol/L, baseline to final, *p* = 0.76; raisins: 1.4 ± 0.2 vs. 1.1 ± 0.2 mmol/L, baseline to final, *p* = 0.24) (Figure 3A).

For HbA1c, there were no differences in the absolute difference from the median either between groups (baseline: 0.7 ± 0.1 vs. 0.6 ± 0.1%, dates vs. raisins, *p* = 0.91; final: 0.7 ± 0.2 vs. 0.6 ± 0.1%, dates vs. raisins, *p* = 0.97) or within groups (dates: 0.7 ± 0.1 vs. 0.7 ± 0.2%, baseline to final, *p* = 0.86; raisins: 0.6 ± 0.1 vs. 0.6 ± 0.1%, baseline to final, *p* = 0.94) (Figure 3B).

## 4. Discussion

This is the first study using date fruit in comparison to a matched glycemic comparator over an intermediate-term, 12-week period. We show that date fruit, at an acceptable level of consumption in a Middle Eastern country, had no effect on glycemic control nor on any of the other cardiovascular risk parameters. In a meta-analysis of five clinical trials, a significant difference was seen in a reduction in fasting plasma glucose [20], a finding not seen here; however, the prior studies that were analyzed were small in nature and conducted over a shorter time period with reportedly high heterogeneity [20]. Here, HbA1c was unaffected, in accordance with a prior study [20]. Given the reported anti-inflammatory and antioxidant actions reported in animal studies [21], we anticipated changes in the inflammatory marker CRP or in lipids, but no such changes were observed. It may be considered that the differences seen between our study and prior reports may be due to the differing properties of the date fruit provided and, whilst that this cannot be excluded, on the basis of reported data, the glycemic indices between different date fruit types do not differ [22]. Others have reported that date fruit may affect glycemic control in animal models [23] and that it has beneficial effects on lipids [24], but replication studies in humans are scant.

Insulin resistance was unchanged in both the dates and the raisins groups over the study period. The consumption of fruit with a low glycemic index and high fiber content, such as raisins, has been shown to decrease insulin resistance in acute studies [25]; however, there was no improvement seen here in insulin resistance (IR), insulin sensitivity (IS) or glycemic control, perhaps because the study was too short in duration for any changes in glycemic control to become evident. In addition, with the concern that the additional prolonged glycemic load may affect the beta cell, there were no changes in beta cell function as determined by HOMA-B or the composite measure of beta cell function, the disposition index. These data show that the consumption of both date fruit and raisins, whilst perhaps not having a beneficial effect on diabetes, at the very least, does not have a detrimental effect on glycemic control.

The mean HbA1c and mean fasting glucose did not differ between or within groups, and therefore, the variability of those parameters was determined. That the variability was found not to differ suggests there would be no impact on microvascular or macrovascular complications [7,8,9]; notably, this is the first study to undertake such an analysis for nutraceutical products.

It was clear that no difference between the raisin and date fruit groups was seen for any of the measured parameters; therefore, the difference in the reported phytoestrogen content was clearly not an important factor, and it is likely that the phytoestrogen levels were too low to effect any changes as was reported in higher phytoestrogen dose studies [15]. Because of the negative results within and between the two groups, phytoestrogens were not measured in the study.

The limitations of this study include that the supplement of the date fruit was not more than 60 g per day, and perhaps a higher supplement would have shown an effect; however, there were no trends seen for any of the parameters, suggesting that a study of longer duration and at much higher fruit quantity above that of normal consumption would still yield an important though negative result. Whilst IR, IS, beta cell function, and glycemic control were not affected, a longer duration of the study or higher date fruit intervention may be thought to reveal differences, but, given that there were no trends in these parameters, this too is unlikely. The study was not blinded, as it would not have been possible to do so, though causes of bias were addressed with the Epic food frequency questionnaire and the IPAQ short last 7-day self-administered questionnaire that accounted for dietary changes and exercise, and these did not differ between groups. Conversely, the major strength of this study was the comparison between matched comparators that had the same glycemic index to determine if one or the other may have additional therapeutic benefits.

In conclusion, date fruit and raisins showed no improvements in insulin resistance, insulin sensitivity, beta cell function, or glycemic control, though it was reassuring that neither 60 g daily of date fruit nor raisins had a deleterious effect on glycemic control over a 12-week period, thereby indicating their safety in T2D; however, no beneficial effects of date fruit on other cardiovascular indices in T2D were seen.

## Figures and Tables

**Table 1:** Demographic and biochemical data for the date fruit (n = 39) and raisins (n = 40) cohorts at baseline. Data presented as mean +/− SD.

Baseline Demographic and Biochemical Data		
Data Presented at Mean +/− SD		
	Dates	Raisins
Age (years)	61 (10)	56 (9)
BMI (kg/m^2^)	31.3 (6.2)	31.3 (5.7)
Systolic BP (mmHg)	133 (20)	133 (17)
Diastolic BP (mmHg)	74 (9)	79 (15)
CRP (mg/L)	7.6 (8.8)	6.6 (5.8)
FBG (mmol/L)	8.1 (2.0)	8.2 (1.7)
HbA1c (%)	7.6 (1.1)	7.7 (0.8)
Fasting insulin (μU/mL)	12.2 (9.2)	12.4 (8.9)
Total cholesterol (mmol/L)	3.9 (1.0)	3.9 (1.0)
Triglycerides (mmol/L)	1.5 (0.7)	1.6 (0.6)
LDL cholesterol (mmol/L)	2.6 (0.9)	2.6 (1.0)
HDL cholesterol (mmol/L)	1.1 (0.2)	1.0 (0.2)
HOMA-IR	4.9 (5.1)	3.8 (3.5)
HOMA-S (%)	39.0 (29.1)	35.0 (20.1)
HOMA-B (%)	61.1 (42.8)	53.7 (35.8)
Disposition index	0.2 (0.4)	0.2 (0.1)

**Table 2:** Demographic and biochemical data both within group and between groups for the date fruit (n = 33 completed) and raisins (n = 28 completed) cohorts after 12 weeks of intervention. Data presented at mean +/− SD.

	Dates			Raisins			Dates vs. Raisins at 12 Weeks
	Baseline	12 weeks	*p*-value	Baseline	12 weeks	*p*-value	*p*-value
BMI (kg/m^2^)	31.3 (6.2)	30.9 (6.4)	0.79	31.3 (5.7)	32.6 (6.1)	0.37	0.27
Systolic BP (mmHg)	133 (20)	128 (20)	0.31	133 (17)	126 (16)	0.12	0.71
Diastolic BP (mmHg)	74 (9)	76 (11)	0.49	79 (15)	79 (8)	0.88	0.20
CRP (mg/L)	7.6 (8.8)	8.3 (8.9)	0.74	6.6 (5.8)	6.0 (4.0)	0.64	0.20
FBG (mmol/L)	8.1 (2.0)	8.7 (2.8)	0.34	8.2 (1.7)	7.8 (1.5)	0.25	0.12
HbA1c (%)	7.6 (1.1)	7.5 (1.1)	0.86	7.7 (0.8)	7.8 (0.8)	0.51	0.72
Fasting insulin (μU/mL)	12.2 (9.2)	9.3 (5.5)	0.12	12.4 (8.9)	9.5 (4.7‘)	0.12	0.91
Total cholesterol (mmol/L)	3.9 (1.0)	4.1 (1.4)	0.48	3.9 (1.0)	3.9 (1.0)	0.9	0.54
Triglycerides (mmol/L)	1.5 (0.7)	1.6 (1.2)	0.80	1.6 (0.6)	1.4 (0.7)	0.22	0.40
LDL cholesterol (mmol/L)	2.6 (0.9)	2.7 (1.4)	0.54	2.6 (1.0)	2.7 (1.0)	0.72	0.95
HDL cholesterol (mmol/L)	1.1 (0.2)	1.1 (0.3)	0.65	1.0 (0.2)	1.0 (0.2)	0.92	0.36
HOMA-IR	4.9 (5.1)	3.8 (3.5)	0.30	4.2 (3.2)	3.2 (1.5)	0.15	0.42
HOMA-S (%)	39.0 (29.1)	41.3 (25.7)	0.73	35.0 (20.1)	41.2 (24.9)	0.28	0.98
HOMA-B (%)	61.1 (42.8)	41.6 (24.7)	0.06	53.7 (35.8)	54.2 (42.4)	0.96	0.17
Disposition index	0.2 (0.4)	0.2 (0.1)	0.31	0.2 (0.1)	0.2 (0.2)	0.30	0.32

## Data Availability

All the data for this study will be made available upon reasonable request to the corresponding author.

## Supplementary Materials

The following supporting information can be downloaded at: https://www.mdpi.com/article/10.3390/nu14173491/s1, Figure S1. Blood glucose comparisons derived from the patient blood glucose profiles using home blood glucose monitoring over the 12-week period of the trial, showing that there were no between or within group changes for either the date fruit or the raisins groups.
