# Supplementary material for: Effect of Date Fruit Consumption on the Glycemic Control of Patients with Type 2 Diabetes: A Randomized Clinical Trial
Source: Nutrients. 2022 Aug 25;14(17):3491. doi: 10.3390/nu14173491 (PMC9458144; doi:10.3390/nu14173491)

Figure Supplementary S1. Blood glucose comparisons derived from the patient blood glucose profiles using home blood glucose monitoring over the 12-week period of the trial, showing that there were no between or within group changes for either the date fruit or the raisins groups.

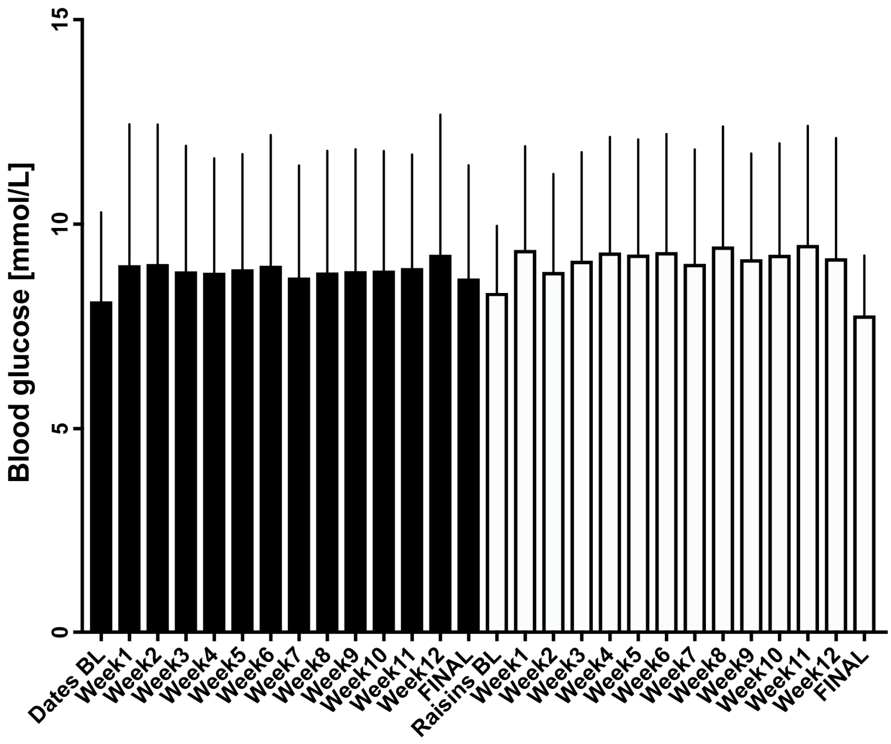

Supplement: Supplementary file 1 [file nutrients-14-03491-s001.zip › nutrients-1842984-supplementary.pdf]
